# Severe Cutaneous Findings in a Woman with Dermatomyositis

**DOI:** 10.5811/cpcem.2019.3.41058

**Published:** 2019-05-20

**Authors:** Sean Wang, Rachel Keaton, Zachary Kendrick

**Affiliations:** University of Texas Health Science Center at San Antonio, Department of Emergency Medicine, San Antonio, Texas

## Abstract

Dermatomyositis is an inflammatory condition characterized by proximal muscle weakness and classic skin manifestations. The severity of these symptoms, however, can vary greatly. Here we present the case of a woman with a particularly severe form of the cutaneous signs. It is important to recognize the potential severity of this condition as the uncontrolled progression of this disease can lead to respiratory compromise and cardiac involvement.

## INTRODUCTION

Dermatomyositis is an idiopathic, inflammatory condition characterized most commonly by proximal muscle weakness and a variety of skin manifestations.^1^ The hallmark dermatologic findings are Gottron’s papules and heliotrope eruptions, but the presence and severity of these findings can vary greatly and do not always correlate with other systemic symptoms.^2^–^3^ Described below is the case of a woman with a particularly severe exacerbation in the setting of recent medication cessation.

## CASE REPORT

A 53-year-old female with a history of heart failure, chronic obstructive pulmonary disease, and biopsy-proven diagnosis of dermatomyositis presented to the emergency department (ED) with a one-week history of worsening shortness of breath, periorbital edema, diffuse pain, and intensely pruritic rash. She reported recent abrupt cessation of her prednisone due to concern for complications related to long-term steroid use to include lumbar spine fracture, as well as methicillin-resistant *Staphylococcus aureus* cellulitis that required intravenous (IV) antibiotics. Regarding her rash, she reported that it had been present since diagnosis of dermatomyositis in 2011; however, the rash had varied in intensity depending on medication regimen and compliance.

On arrival, the patient was in moderate distress secondary to pain but was speaking in full sentences. On presentation her vital signs were as follows: afebrile at 98.4 degrees Fahrenheit, tachycardic to 108 beats per minute, respiratory rate 18 beats per minute, oxygen saturation 100% on room air, blood pressure 138/73 millimeters of mercury, and reported 10/10 pain diffusely, but worse on face and scalp. Physical exam was significant for severe rash with erythematous and violaceous macules and patches of excoriation and lichenification over her scalp, face, neck, chest, abdomen, back, and on dorsal surface of arms, with the worst areas on her scalp (Image). Her exam was notable for severe excoriations of the scalp resulting in serosanguinous drainage. There was significant periorbital edema resulting in difficulty opening her eyes voluntarily. The skin on her arms was sclerosed, making it difficult to obtain dependable vascular access, eventually necessitating placement of a central venous catheter in her right internal jugular. She had mild contractures at both elbows bilaterally and was unable to fully extend her arms. Her lower extremities were notable for 1+ non-pitting edema to her knees. Her abdomen was soft and non-tender, and although the rash was present on her abdomen, the skin was not as sclerosed as the scalp, face, and extremities. The rest of her physical exam was relatively normal with clear lung sounds, no abnormal heart sounds, and an unremarkable neurological exam.

The patient was given 125 milligrams IV methylprednisolone, one liter (L) bolus of normal saline, and pain control medication. Laboratory results revealed alkaline phosphatase 246 international units per liter (IU/L), B-type natriuretic peptide 585 picogram/milliliter, hemoglobin 7.8 grams per deciliter, hematocrit 26.9%, and erythrocyte sedimentation rate 54 millimeters per hour. The remaining laboratory values were as detailed in the Table.

The patient was admitted and remained in the hospital for seven days. She received a course of IV steroids, and we obtained repeat punch biopsies. The biopsies demonstrated thickening of the basement membrane with dermal fibrosis, superficial vascular ectasia, and underlying septal-predominant panniculitis – features consistent with dermatomyositis. Dermatology and rheumatology follow-up appointments were made for the patient prior to discharge, but as of writing this report the patient had not kept her appointments and was not answering phone calls. Attempts are still being made to contact this patient for follow-up and continuation of treatment.

## DISCUSSION

According to a database review study out of Ontario, Canada, only 3.3% of ED visits over a five-year period presented with a primary dermatologic complaint – approximately half of which were for a soft tissue infection rather than autoimmune pathologies. Of that 3.3%, only 4% required inpatient admission, making severe dermatologic conditions a relatively rare occurrence in the ED.^4^

When evaluating patients who present to the ED with a diffuse rash such as found in this case, many other conditions must be considered. Patients with systemic lupus erythematosus (SLE) can also present with facial erythema and photosensitive eruptions. SLE, however, will often spare the nasolabial folds and lacks the pronounced muscular involvement classically seen with dermatomyositis.^5^ Scalp dermatomyositis can also appear similar to cutaneous findings seen in psoriasis. However, psoriasis does not exhibit the poikilodermatous changes typically present in dermatomyositis, a characteristic exemplified in the case presented here.^6^ One must also consider infectious etiologies or, of more concern, a bacterial infection superimposed on a pre-existing autoimmune rash. In this case, while it was impossible to differentiate erythema caused by her chronic skin condition from erythema that could indicate infection, there were no other findings concerning for a concurrent infection (i.e., leukocytosis, fever, hypotension).

**CPC-EM Capsule**

What do we already know about this clinical entity?

Dermatomyositis is an autoimmune condition with a wide spectrum of severity. Most treatments only manage symptoms, attempt to slow the progression, and prevent complications.

What makes this presentation of disease reportable?

We present a case of dermatomyositis of particular severity that demonstrates the importance of helping patients overcome obstacles to compliance.

What is the major learning point?

Dermatomyositis, in severe cases, can produce significant scarring, pain, and risk of infection.

How might this improve emergency medicine practice?

Providers need to recognize the spectrum of presentations, enabling us to both treat exacerbations and provide education and follow-up to help patients long-term.

Dermatomyositis is a rare condition that affects an estimated two in 100,000 annually in the general population and has a 2:1 female predominance.^7^ The exact pathogenesis is not fully understood, but much of the literature demonstrates type 1 interferons and/or antibody and complement mediated damage to myofibrils and capillaries.^8^–^10^ Research into the development of the condition is made difficult by the low incidence of the disease. The paucity of large, randomized control studies makes standardized, targeted therapy especially difficult; most treatment regimens still focus on high-dose glucocorticoids and immunosuppressants.

Contributing to the confusion surrounding the disease is the variable expression of symptoms among patients. The severity of the rash will vary from Gottron’s papules to a generalized erythroderma with variation presumably attributed to protective factors, genetic allotypes, and how quickly treatment was initiated after diagnosis.^11^–^12^ Clinical features that have been associated with a worse outcome include the following: delay in initiation of treatment for more than six months after onset of symptoms; significant muscular weakness at presentation; dysphagia; respiratory muscle weakness; interstitial lung disease; cardiac involvement; and associated malignancy.^13^–^17^ Despite the need for more information and the variable presentation, increased recognition and rapid initiation of treatment has led to an increased rate of remission and an improved five-year mortality for these patients. From 1971 to 1985, the five-year survival rate for inflammatory myopathies was 52–65%, but improved to 75–95% in studies from 2001 to 2006. Among the causes of death in this population, the most common are malignancies, infection, respiratory failure, and cardiovascular disease.^18^

The case of the patient presented here was a particularly serious one. Of the known features associated with poor prognosis, our patient demonstrated almost all of them. While she seemed to have been started on treatment soon after diagnosis in 2011, she had, at best, intermittent treatment due to long periods of non-compliance. Muscular weakness was not a predominant feature at the time of this presentation, but the presence of contractures in her bilateral upper extremities and a documented history of proximal upper extremity weakness were concerning for a poor prognosis. She also had a documented history of dysphagia with repeated neurologic evaluation. These factors combined with her existing comorbidities placed her at significant risk for poor outcome.

## CONCLUSION

Dermatomyositis is a rare, incompletely-understood condition that presents with characteristic cutaneous findings. The patient discussed here presented with a severe form of the rash. It is likely that the severity of her condition was due in significant part to poor compliance and other social issues that prevented her from participating in treatment. Given the wide spectrum of severity seen in dermatomyositis, there remains much work to discover effective, affordable treatment. While not a common occurrence in the ED, it is still of vital importance for emergency physicians to recognize dermatomyositis exacerbations when they present. Given that it is a progressive disease, delays in treatment or misdiagnosis can lead to devastating changes that affect quality of life (scarring, expensive therapies, etc.), and can increase risk of death.

## Figures and Tables

**Image:**
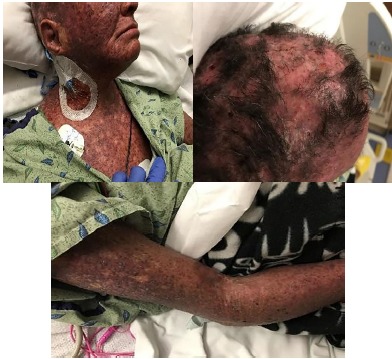
Severe excoriations and rash over upper chest, scalp, and arm respectively.

**Table:** Initial laboratory results for patient with dermatomyositis.

Lab	Result
Complete blood count	
White blood cell	7.35 K/mcL
Hemoglobin	7.8 g/dL
Hematocrit	26.9 %
Platelets	469 K/mcL
Complete metabolic panel	
Sodium	137 mmol/L
Potassium	3.2 mmol/L
Chloride	102 mmol/L
Carbon dioxide	30 mmol/L
Blood urea nitrogen	7 mg/dL
Creatinine	0.38 mg/dL
Glucose	132 mg/dL
Liver	
Aspartate aminotransferase	48 U/L
Alanine aminotransferase	19 U/L
Alkaline phosphatase	246 U/L
Bilirubin (total)	0.6 mg/dL
Albumin	2.0 g/dL
Cardiac	
B-type natriuretic peptide	585 pg/ml
Troponin	<0.030 ng/ml
Other	
Creatine kinase	53 U/L
Lactic acid	1.6 mmol/L
Lipase	259 U/L
Erythrocyte sedimentation rate	54 mm/hr

*K/mcL,* thousands per microliter; *g/dL,* grams per deciliter; *mmol/L,* millimoles per liter; *mg/dL,* milligram per deciliter; *U/L,* units per liter; *pg/ml,* picograms per milliliter; *ng/ml,* nanograms per milliliter; *mm/hr,* millimeter per hour.
